# Vitamin D3 Levels in Predicting Transient Hypocalcemia After Parathyroidectomy

**DOI:** 10.7759/cureus.26576

**Published:** 2022-07-05

**Authors:** Chirag Pereira, Benji Varghese

**Affiliations:** 1 General Surgery, Lancaster Royal Infirmary, Lancaster, GBR; 2 Urology, Wythenshawe Hospital, Manchester, GBR

**Keywords:** neck endocrine surgery, postoperative hypocalcemia, 25-oh vitamin d, minimally invasive parathyroidectomy, parathyroid hormone (pth)

## Abstract

Introduction

Hypocalcemia following parathyroidectomy can often be problematic, requiring long-term follow-up. This complication can be permanent or transient. It most commonly occurs following head and neck surgery where there is accidental removal of the parathyroid gland or damage to the blood supply to the gland during dissection. This study aimed to predict transient hypocalcemia and hypoparathyroidism by evaluating preoperative vitamin D.

Methods

We retrospectively reviewed the medical records of 82 patients that had undergone minimally invasive surgery for primary hyperparathyroidism. Data for patient demographics, histopathology, preoperative and postoperative parathyroid hormone (PTH), calcium levels and vitamin D levels were reviewed.

Results

The female to male ratio was 8.1:1 and the mean age was 56.4 ± 6.2. Preoperative vitamin D was normal in 47.6%, 39% had vitamin D deficiency and 13.4% had vitamin D insufficiency. Postoperatively, 23% has hypocalcemia and 10% had hypoparathyroidism. Postoperative median calcium levels were low in both the vitamin D deficiency and insufficiency groups but failed to show a significant association.

Conclusion

In our study, preoperative vitamin D levels failed to show an association between postoperative calcium and PTH levels.

## Introduction

A complex process is involved in maintaining calcium homeostasis in the human body where three key components, that is, serum calcium, serum phosphate 1,25-dihydroxy vitamin D-3, and parathyroid hormone (PTH) play a key role. Vitamin D-3 is synthesised in the skin from cholesterol precursors when the skin is exposed to sunlight. It further undergoes hydroxylation in the liver and kidney to form 1,25-dihydroxy vitamin D-3, which is the active metabolite of vitamin D-3 and plays an important role in the absorption of calcium and phosphate from the gastrointestinal tract [1].

PTH is synthesized from parathyroid glands in the neck in response to low levels of circulating serum calcium. They act principally on three main sites: the skeletal system, gastrointestinal tract and kidneys. This promotes the absorption of calcium in the kidneys; in the bones, it promotes osteoclastic activity; and in the intestine, it promotes the absorption of calcium. Primary hyperparathyroidism (pHPT) results from excessive production of parathyroid hormone from one or more abnormal parathyroid glands. The net result of primary hyperparathyroidism is high circulating levels of serum calcium resulting in symptoms and signs of hypercalcemia. The most common cause of primary hyperparathyroidism is a solitary adenoma followed by parathyroid hyperplasia and, rarely, parathyroid carcinoma [2].

Medical management of parathyroid disease is mainly aimed at controlling hypercalcemia and providing symptomatic relief. Definitive management of parathyroid adenoma is surgery, which is either done as standard neck exploration or minimally focused surgery. One of the common postoperative complications following parathyroidectomy or even thyroid surgery is hypocalcemia [3]. This can be either transient or permanent. Transient hypocalcemia is believed to result from intraoperative damage to the parathyroid gland or disruption of blood supply to these glands during surgery [4]. Although there is no clear definition of transient hypocalcemia, most studies consider a six-month cut-off post-surgery to distinguish transient from permanent postoperative hypocalcemia [5-6].

The aim of our study was to evaluate if preoperative vitamin D deficiency could be a risk factor for postoperative hypocalcemia and hypoparathyroidism following surgery for pHPT.

## Materials and methods

The medical records of patients who underwent parathyroidectomy at Royal Lancaster Infirmary for symptomatic hyperparathyroidism were reviewed from January 2019 to December 2021. Inclusion criteria included patients that underwent unilateral parathyroidectomy via a minimally invasive approach. The diagnosis was confirmed by intraoperative PTH monitoring and histopathology. Patients with permanent hypoparathyroidism, renal impairment, multiple gland disease, hereditary disease, taking medications like diuretics, vitamin D or calcium supplementation, thyroidectomy performed jointly with parathyroidectomy and incomplete medical records were excluded from the study. All operations were performed by two senior endocrine surgeons.

The following data were collected from medical records: age, sex and histopathology reports and preoperative corrected calcium and vitamin D-3 levels. Serum-corrected calcium and PTH levels were checked on the first postoperative day and one month following surgery. Normal calcium levels ranged from 2.20 to 2.55 mmol/L, PTH levels ranged from 1.3 -9.3 pmol/L and vitamin D levels greater than 50 nmol/L were normal. Vitamin D deficiency was defined as levels less than 50 nmol/L while vitamin D insufficiency ranged from 50 to 75 nmol/L. In our study, transient hypocalcemia was accepted as corrected calcium less than 2.20 mmol/L until one-month postoperatively. Since this is a retrospective review, informed consent was not required.

Statistical analysis

Categorical data were presented in the form of frequencies and percentages while continuous data were presented as mean ± standard deviation and non-normally distributed data were represented as median values. The normality of data distribution was confirmed by the Kolmogorov-Smirnov test. The chi-square test was used to determine the relationship between categorical variables, and a p-value of less than 0.05 was considered statistically significant. Statistical analysis was performed using IBM SPSS for Mac version 28 (IBM Corp., Armonk, NY).

## Results

A total of 114 medical records were reviewed over a period of three years. We had to exclude 32 cases from the study, as nine had incomplete medical records, two patients underwent simultaneous thyroidectomy with parathyroid surgery, four patients had renal impairment and 17 patients were on medications (calcimimetics, vitamin D supplementation and diuretics), leaving behind 82 cases. There were 62 (75.6%) females and 20 (24.4%) males in the study with the mean age being 62.6 ± 5.6 years. Demographic and biochemical parameters are given in Table 1.

**Table 1:** Clinical and Demographic Data Before the Surgery PTH: parathyroid hormone

N	82
Age (years)	56.4 ± 6.2
Female / Male	63 (76.8%) / 9 (10.9%)
Serum calcium (mmol/Lt)	2.9 ± 0.4
PTH (pmol/Lt)	15.9 ± 7.9
25-Hydroxy Vitamin D3 (nmol/Lt)	57.2 ± 19.5

**Figure 1:**
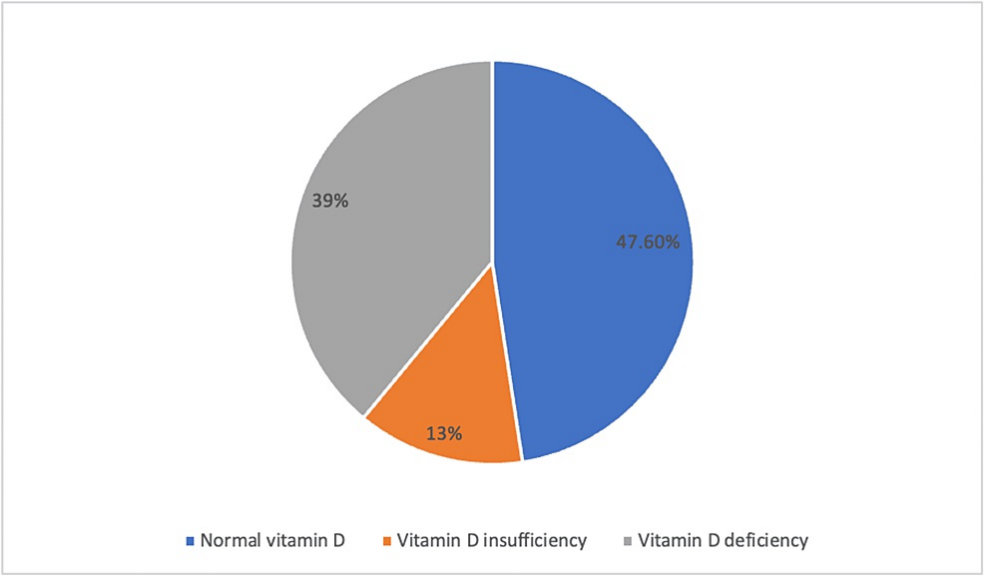
Preoperative Vitamin D Status

Based on histopathology, adenoma was noted in 51 cases (62.1%) and parathyroid hyperplasia in 63 cases (76.8%). We did not have cases of parathyroid malignancies. Preoperative vitamin D levels were normal in 39 (47.6%) cases, 32 (39%) cases had vitamin D deficiency and 11 (13.4%) cases had vitamin D insufficiency (Figure 1).

At the end of the study, transient hypocalcaemia was noted in 27 patients (23%) and transient hypoparathyroidism was noted in 11 patients (10%). Following parathyroidectomy, median serum corrected calcium levels at one month postoperatively were low in both vitamin D deficiency and insufficiency groups in comparison to normal vitamin D level groups but failed to show a statistical significance (P=0.61). Median levels of serum PTH following thyroidectomy were similar in all the vitamin D groups (Table 2).

**Table 2:** Median Postoperative Serum Calcium and PTH Levels in Relation to Vitamin D Levels PTH: parathyroid hormone

	Vitamin D levels			
	>75 nmol/L	50-75 nmol/L	<50 nmol/L	p-value
Calcium (mmol/Lt)	2.43 (2.1-3.01)	2.31 (2-2.51)	2.33 (2.1-2.51)	0.61
PTH (pmol/Lt)	4.1 (2-6.1)	4.1 (2.3-6.1)	6.1 (3.2-8.5)	0.21

The percentage of transient hypocalcaemia and hypoparathyroidism was higher in the vitamin D insufficiency and deficiency group but failed to show a statistical significance (Table 3). At six months of follow-up, all patients in this study had normal levels of serum calcium.

**Table 3:** Comparison Among Three Vitamin D Groups and Transient Hypocalcemia and Hypoparathyroidism

	Vitamin D levels			
	>75 nmol/L	50-75 nmol/L	<50 nmol/L	p-value
Transient hypocalcaemia (%)	22.2	40.7%	37%	0.12
Transient hypoparathyroidism (%)	18.1%	36.3%	45.4	0.14

## Discussion

The most common cause of pHPT is a single parathyroid adenoma and the management of these tumours has most commonly been the surgical approach. Indications for surgery include age younger than 50 years, creatinine clearance <60 ml/min, 24-hour urinary calcium excretion >400 mg/day, evidence of vertebral fracture on X-ray, serum calcium > 0.05 mmol/L above the upper limit of the reference range and bone mineral density T-score at or below 2.5 [7]. The most common approach in the past has been standard bilateral neck exploration but with the advancement in preoperative localization and intraoperative PTH monitoring, a minimally invasive or focused approach to parathyroidectomy is more commonly being performed. Most common indications for surgery include symptomatic hypercalcemia, osteoporosis, nephrocalcinosis and renal insufficiency [8]. A meta-analysis by Qin et al. found that young age, female sex, parathyroid auto-transplantation, inadvertent parathyroid excision, Graves’ disease, thyroid cancer, central lymph node dissection, preoperative Vitamin D deficiency and lower postoperative 24-hour PTH levels were risk factors for transient hypocalcemia [6].

Primary hyperparathyroidism is a disease that more commonly affects women. In our study, there were more women as compared to men with a ratio of 8.1:1. The age of patients ranged from 24 to 89 years. Hypocalcemia is a complication following head and neck surgery resulting from inadvertent removal of the parathyroid gland or damage to the blood supply of the gland, the risk of which is greatly increased in cases of total thyroidectomy or bilateral cervical lymph node dissection [9]. Hypocalcemia can be transient or permanent. Most authors believe that transient hypocalcemia resolves within six months; if not, patients are termed to have permanent hypocalcemia, which is a result of poor parathyroid reserve [5-6]. In our study, we only included patients who had undergone unilateral parathyroidectomy by a focused approach. A systemic review and meta-analysis by Jinih et al. found that the rate of postoperative hypocalcemia following focused parathyroidectomy was 1.6% versus 13.2% in bilateral neck exploration [10].

Vitamin D plays an important role in calcium homeostasis along with PTH. Its main mechanism of action is in the intestine, where it promotes the uptake of calcium from the gut. Following parathyroidectomy, PTH levels drop down quite drastically and this promotes increased calcium uptake into bones and reduced bone resorption. Hypocalcemia can also occur due to the loss of PTH-mediated activation of vitamin D into calcitriol, which is its active form [11]. Our study failed to show a significant association between preoperative vitamin D and transient hypocalcemia. Similar to our study, Falcone et al. and Soares et al. failed to show an association between vitamin D and postoperative hypocalcemia [12-13]. Soares et al. did, however, show that hypocalcemia is dependent on postoperative levels of PTH [13]. Unsal et al., on the other hand, in their study, showed that there was a significant drop in postoperative calcium levels following parathyroidectomy in patients with vitamin D deficiency and vitamin D insufficiency [14].

The main limitation of our study is that it is a retrospective study. We reviewed patient data upto six months following their surgery but did not look for recurrent hypercalcemia beyond this period, which could have arisen from a new or missed parathyroid adenoma.

## Conclusions

Parathyroidectomy plays an important role in managing primary hyperparathyroidism. Our study failed to show an association between preoperative vitamin D levels and postoperative serum calcium levels. There are very limited data available showing the relationship between preoperative vitamin D and its effect on postoperative calcium levels. More prospective studies with a larger data population are needed to evaluate this relationship.
